# Early Pregnancy Loss Management in the Emergency Department vs Outpatient Setting

**DOI:** 10.1001/jamanetworkopen.2023.2639

**Published:** 2023-03-15

**Authors:** Lyndsey S. Benson, Sarah K. Holt, John L. Gore, Lisa S. Callegari, Anne K. Chipman, Larry Kessler, Vanessa K. Dalton

**Affiliations:** 1 Department of Obstetrics and Gynecology, University of Washington School of Medicine, Seattle; 2 Department of Urology, University of Washington School of Medicine, Seattle; 3 US Department of Veterans Affairs Puget Sound Health Care System, Health Services Research and Development, Seattle, Washington; 4 Department of Health Systems and Population Health, University of Washington School of Public Health, Seattle; 5 Department of Emergency Medicine, University of Washington School of Medicine, Seattle; 6 Department of Obstetrics and Gynecology, University of Michigan, Ann Arbor

## Abstract

**Importance:**

Early pregnancy loss (EPL), or miscarriage, is the most common complication of early pregnancy, and many patients experiencing EPL present to the emergency department (ED). Little is known about how patients who present to the ED with EPL differ from those who present to outpatient clinics and how their management and outcomes differ.

**Objective:**

To compare the management and outcomes of patients with EPL who present to the ED vs outpatient clinics.

**Design, Setting, and Participants:**

This retrospective cohort study used the IBM MarketScan Research Database, a national insurance claims database. Participants were pregnant people aged 15 to 49 years in the US who presented to either an ED or outpatient clinic for initial diagnosis of EPL from October 2015 through December 2019. Data analysis was performed from May 2021 to March 2022.

**Exposures:**

The primary exposure was location of service (ED vs outpatient clinic). Other exposures of interest included demographic characteristics, current pregnancy history, and comorbidities.

**Main Outcomes and Measures:**

The primary outcome was EPL management type (surgical, medication, or expectant management). Complications, including blood transfusion and hospitalization, and characteristics associated with location of service were also evaluated. Bivariable analyses and multivariable logistic regression were used for data analysis.

**Results:**

A total of 117 749 patients with EPL diagnoses were identified, with a mean (SD) age of 31.8 (6.1) years. Of these patients, 20 826 (17.7%) initially presented to the ED, and 96 923 (82.3%) presented to outpatient clinics. Compared with the outpatient setting, patients in the ED were less likely to receive surgical (2925 patients [14.0%] vs 23 588 patients [24.3%]) or medication (1116 patients [5.4%] vs 10 878 patients [11.2%]) management. In the adjusted analysis, characteristics associated with decreased odds of active (surgical or medication) vs expectant management included ED (vs outpatient) presentation (adjusted odds ratio [aOR], 0.46; 95% CI, 0.44-0.47), urban location (aOR, 0.87; 95% CI, 0.82-0.91), and being a dependent on an insurance policy (vs primary policy holder) (aOR, 0.71; 95% CI, 0.67-0.74); whereas older age (aOR per 1-year increase 1.01; 95% CI, 1.01-1.01), established prenatal care (aOR, 2.35; 95% CI, 2.29-2.42), and medical comorbidities (aOR, 1.05; 95% CI, 1.02-1.09) were associated with increased odds of receiving active management. Patients in the ED were more likely than those in outpatient clinics to need a blood transfusion (287 patients [1.4%] vs 202 patients [0.2%]) or hospitalization (463 patients [2.2%] vs 472 patients [0.5%]), but complications were low regardless of location of service.

**Conclusions and Relevance:**

In this cohort study of privately insured patients with EPL, differences in management between the ED vs outpatient setting may reflect barriers to accessing comprehensive EPL management options. More research is needed to understand these significant differences in management approaches by practice setting, and to what extent EPL management reflects patient preferences in both outpatient and ED settings.

## Key Points

**Question:**

Do management and outcomes of early pregnancy loss (EPL) differ for patients who present to an emergency department (ED) vs those who present to an outpatient clinic?

**Findings:**

In this cohort study of 117 749 patients with EPL, patients who presented to the ED were less likely to receive surgical or medication management than patients presenting to an outpatient clinic.

**Meaning:**

These findings suggest that patients with EPL diagnosed in the ED vs outpatient clinics are less likely to receive active management via surgery or medication; these differences may reflect barriers to accessing comprehensive EPL management options.

## Teaser

This cohort study compares the management and outcomes of patients with early pregnancy loss who present to the emergency department vs outpatient clinics.

## Introduction

Early pregnancy loss (EPL), or miscarriage, is the most common pregnancy complication.^1,2,3,4^ Up to 20% of pregnancies end in EPL, which is defined as a nonviable, intrauterine pregnancy within the first 12 weeks of gestation.^1^ Although many patients seek care for EPL in the outpatient setting, a substantial portion present to the emergency department (ED) for a variety of reasons, including lack of an established physician, ease of access, or perceived sense of urgency.^5,6^ EPL or bleeding in early pregnancy accounts for a combined 2.7% of all ED visits among reproductive-aged women, or approximately 900 000 ED visits annually.^7^

Treatment options for patients with a diagnosis of EPL include expectant management, medication management, or surgical management via uterine aspiration.^1^ Although these management options are all typically available to patients who present to an outpatient clinic, it is unclear which options are routinely offered to patients who initially present to an ED.^8,9^

Multiple studies^5,10^ have demonstrated that patient experiences of EPL in the ED setting differ from those in the outpatient clinic setting. Patients who seek EPL care in the ED report that they do not receive adequate communication, follow-up, or emotional support, and they may not have the same access to active (either surgical or medication) management as patients seen in the outpatient setting.^5,11,12,13,14^ Delays in treatment and inadequate communication exacerbate the psychological trauma associated with EPL.^15,16,17^ The factors associated with presentation to the ED vs the outpatient setting, and the ways in which management differs in these settings, have not been well researched.

A substantial portion of patients with EPL present to the ED for management, but little is known about how these patients differ from those who present to an outpatient clinic.^7^ Furthermore, little is known about how EPL management in the ED differs from the outpatient setting. A better understanding of how EPL presentation and management differs between the ED and outpatient clinics could inform future efforts to improve clinical care and patient experiences with EPL in the ED setting.

We sought to identify characteristics associated with type of EPL management (surgical, medication, and expectant management) using a national claims database. We also sought to describe characteristics of patients presenting with EPL to the ED vs outpatient setting, and to compare clinical outcomes and complications by location of service. We hypothesized that patients presenting with EPL to the ED would be less likely to undergo active management of EPL within a week following their diagnosis compared with patients presenting to outpatient settings.

## Methods

### Study Design and Data Source

We performed a retrospective cohort study of pregnant people aged 15 to 49 years who experienced EPL in the US from October 2015 to December 2019 using the IBM MarketScan Research Database. MarketScan comprises deidentified claims data for more than 273 million privately insured individuals. The MarketScan data represent closed and adjudicated claims obtained from a large convenience sample of employers and health plans that agree to participate. Available data include dates and locations of service, demographic characteristics, diagnosis and procedure codes, and medication prescriptions.

This study was exempt from review by the University of Washington institutional review board. We followed the Strengthening the Reporting of Observational Studies in Epidemiology (STROBE) reporting guideline for cohort studies. Informed consent was not needed because the data were deidentified, in accordance with 45 CFR §46.

### Study Population

Eligible patients had available data for 90 days before and 60 days after the index EPL diagnosis, had prescription data available, and had their initial diagnosis of EPL in either an ED or outpatient clinic. For patients with recurrent pregnancy losses, only the first EPL episode was included in this analysis. We excluded patients who had a concurrent diagnosis of ectopic or molar pregnancy, induced abortion, or stillbirth, or who had evidence of recent EPL management (either with medication or uterine aspiration). To exclude patients who likely had a pregnancy diagnosis other than EPL, we used the validated algorithm developed by Matcho et al^18^ to infer pregnancy episodes and outcomes. Patients who had evidence of recent EPL management were excluded if they had a prescription for misoprostol or underwent uterine aspiration within 56 days before their index EPL diagnosis; this was the minimum length of time to be considered a separate pregnancy episode per the algorithm by Matcho et al.^18^ We also excluded patients who received care at both an ED and outpatient setting on the date of their index diagnosis, because it was not possible to determine initial location of service.

We identified EPL diagnoses with the following *International Classification of Diseases, Tenth Revision, Clinical Modification (ICD-10-CM)* diagnosis codes: O02.1 (missed abortion) and O03.x (spontaneous abortion) (eTable 1 in Supplement 1). The study time period was chosen to coincide with the transition to *ICD-10-CM* codes.

### Exposures

The primary exposure of interest was location of service (ED vs outpatient setting). Location of service was determined by the MarketScan place-of-service code variable (stdplac) coded as either 23 (emergency room) or 11 (office), and other locations of service were not included.^19^

Additional variables or covariates of interest included age (in years), insurance policy status (primary policy holder or spouse vs dependent), metropolitan statistical area status (rural vs urban location), geographic region (West, Northeast, North-Central, and South), type of insurance plan (health maintenance organization, preferred provider organization, exclusive provider organization, or other), and year of index EPL diagnosis. We examined whether patients had received prenatal care before their EPL diagnosis and whether they had a prior diagnosis of threatened EPL in the current pregnancy. Threatened EPL typically refers to a pregnancy affected by bleeding or cramping without a definitive diagnosis of miscarriage. We also included a history of infertility diagnosis or treatment. Finally, we used the Elixhauser Comorbidity Index to control for comorbidities.^20^ We used a binary score of 1 or more of 30 comorbidities listed in the Elixhauser index.^19^ All covariates included in regression analyses were selected a priori on the basis of prior knowledge and clinical relevance.

### Outcomes

The primary outcome was type of EPL management. We categorized patients as receiving surgical management if they underwent a uterine aspiration procedure at any location within 7 days of index EPL diagnosis (eTable 1 in Supplement 1). Of those remaining, patients were categorized as receiving medication management if they received either mifepristone or misoprostol within 7 days. We categorized patients as undergoing expectant management if they received neither surgical nor medication management within 7 days following their index EPL diagnosis. We used the term *active management* to refer to patients who received either surgical or medication management within 7 days of EPL diagnosis.

Secondary outcomes included hospital admission and complications that occurred in the following 6 weeks. We examined hospitalizations related to EPL, as well as hospitalizations for all indications. Complications included repeat uterine aspiration (for patients who already underwent 1 uterine aspiration), hemorrhage requiring blood transfusion, uterine artery embolization, hysterectomy or other surgical management, cervical laceration, uterine perforation or other genitourinary tract injury, and intrauterine infection. Because of the low rates of complications overall, we created a composite complication variable that included hospitalization related to EPL, blood transfusion, and hysterectomy or other surgical management (apart from uterine aspiration). All outcomes and complications were determined through *ICD-10-CM* diagnosis codes, *ICD-10-CM* procedure codes, and *Current Procedural Terminology* codes (eTable 1 in Supplement 1).

### Statistical Analysis

Data analysis was performed from May 2021 to March 2022. We conducted a descriptive analysis of management type by location of service (ED vs outpatient setting). Using bivariable and multivariable analyses, we identified factors associated with ED vs outpatient presentation. We performed bivariable analyses using χ^2^ tests for categorical variables and *t* tests for continuous measures comparing ED vs outpatient encounters, with 2-tailed *P* < .05 considered to denote statistical significance. Multivariable logistic regression was used to determine the factors associated with presentation at the ED vs outpatient setting. Multivariable logistic regression was also used to determine the odds of active treatment (with surgical or medication management) for patients seen in the ED vs outpatient clinic setting.

We performed descriptive statistics of complications and conducted bivariable analyses comparing complications between the ED and outpatient cohorts and between management types. We performed multivariable logistic regression to determine characteristics associated with the composite complication outcome. We planned a priori to conduct a sensitivity analysis in which we included the patients who had both ED and outpatient encounters on the index date of EPL diagnosis with either the ED cohort or the outpatient cohort. All statistical analyses were conducted in SAS statistical software version 9.4 (SAS Institute).

## Results

We identified 117 749 patients, with a mean (SD) age of 31.8 (6.1) years, who received an index diagnosis of EPL in either an ED or outpatient setting from October 2015 through December 2019 (Figure). Our initial cohort included 147 035 patients aged 15 to 49 years, and we excluded 29 286 patients who met our exclusion criteria, including 975 who received care at both an ED and outpatient setting on the same day. Of 117 749 remaining patients, 20 826 (17.7%) received their initial care in the ED and 96 923 (82.3%) received their initial care in an outpatient setting.

**Figure.**
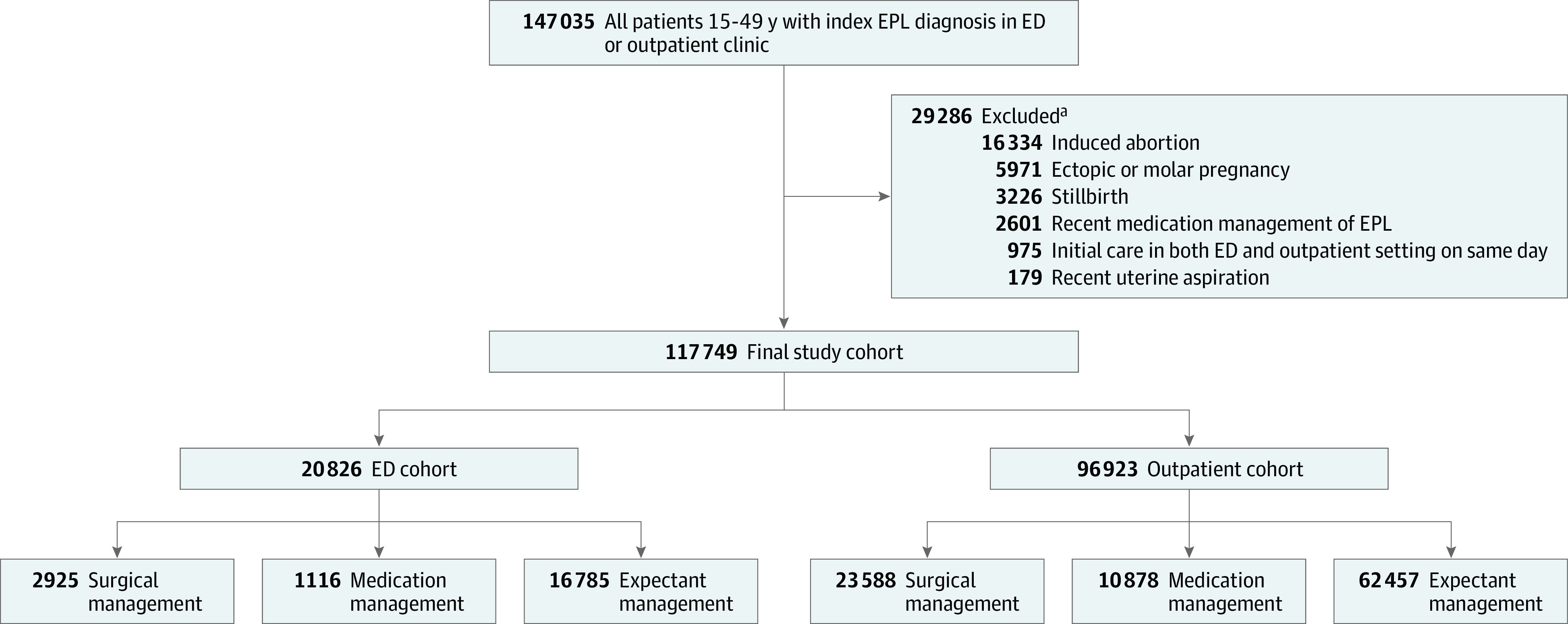
Patient Selection Flowchart ED indicates emergency department; EPL, early pregnancy loss. ^a^Exclusions were performed in this order of priority and these numbers are mutually exclusive. The final exclusion criterion (“Initial care in both ED and outpatient setting on same day”) was performed last, and sensitivity analyses were conducted without applying this exclusion criterion.

Patients who received their initial EPL diagnosis in an ED were younger than the outpatient clinic cohort, with a mean (SD) age of 29.6 (6.7) vs 32.2 (5.9) years (Table 1). The proportion of patients who were at least 35 years old (ie, advanced maternal age), was 24.7% (5139 patients) in the ED group, compared with 36.0% (34 887 patients) in the outpatient group. A total of 4994 patients in the ED cohort (22.1%) were a dependent on their insurance plan, rather than the primary policy holder or spouse, compared with 8303 (8.6%) in the outpatient group. The proportion of patients presenting initially to the ED increased each year, from 15.9% (1401 of 8778 patients) in 2015 to 18.4% (4282 of 23 258 patients) in 2019. More than one-fourth of patients in the ED cohort (5261 patients [25.3%]) had at least 1 medical comorbidity, compared with 15 482 patients (16.0%) in the outpatient cohort.

**Table 1.** Demographic Characteristics of Patients With Early Pregnancy Loss Who Presented to an ED or Outpatient Clinic

Characteristic	Participants, No. (%)			*P* value
	Total (N = 117 749)	ED cohort (n = 20 826)	Outpatient cohort (n = 96 923)	
Age, mean (SD), y	31.8 (6.1)	29.6 (6.7)	32.2 (5.9)	<.001
Age category, y				
15-19	2659 (2.3)	1109 (5.3)	1550 (1.6)	<.001
20-24	13 327 (11.3)	4411 (21.2)	8916 (9.2)	
25-29	25 135 (21.4)	5002 (24.0)	20 133 (20.8)	
30-34	36 602 (31.1)	5165 (24.8)	31 437 (32.4)	
35-39	27 359 (23.2)	3491 (16.8)	23 868 (24.6)	
40-49	12 667 (10.8)	1648 (7.9)	11 019 (11.4)	
Encounter year				
2015^a^	8778 (7.5)	1401 (6.7)	7377 (7.6)	<.001
2016	29 872 (25.4)	5131 (24.6)	24 741 (25.5)	
2017	27 476 (23.3)	4886 (23.5)	22 590 (23.3)	
2018	28 365 (24.1)	5126 (24.6)	23 239 (24.0)	
2019	23 258 (19.8)	4282 (20.6)	18 976 (19.6)	
Insurance coverage				
Primary or spouse	104 852 (89.1)	16 232 (77.9)	88 620 (91.4)	<.001
Dependent	12 897 (11.0)	4994 (22.1)	8303 (8.6)	
Insurance plan type				
Preferred provider organization or exclusive provider organization	61 390 (52.1)	10 610 (51.0)	50 780 (52.4)	<.001
Health maintenance organization	12 779 (10.9)	2254 (10.8)	10 525 (10.9)	
Other	43 580 (37.0)	7962 (38.2)	35 618 (36.8)	
Metropolitan statistical area				
Urban	110 146 (93.5)	19 637 (94.3)	90 509 (93.4)	<.001
Rural	7603 (6.5)	1189 (5.7)	6414 (6.6)	
Geographic region^b^				
Northeast	20 224 (17.2)	2732 (13.1)	17 492 (18.1)	<.001
North-Central	22 511 (19.1)	4150 (19.9)	18 361 (18.9)	
South	54 597 (46.4)	10 840 (52.1)	43 757 (45.2)	
West	20 268 (17.2)	3069 (14.7)	17 199 (17.8)	
Established prenatal care in current pregnancy	38 509 (32.7)	5965 (28.6)	32 544 (33.6)	<.001
Threatened early pregnancy loss in current pregnancy	40 149 (34.1)	6427 (30.9)	33 722 (34.8)	<.001
History of infertility diagnosis or treatment	7262 (6.2)	444 (2.1)	6818 (7.0)	<.001
History of comorbidity	20 743 (17.6)	5261 (25.3)	15 482 (16.0)	<.001

Abbreviation: ED, emergency department.

^a^ The study period was from October 2015 through December 2019, so 2015 proportions are smaller because there are data from only 3 months in 2015.

^b^ Proportions by geographic area do not add up to 100% because of missing data (0.13% unknown geographic region); this was the only variable with missing data.

In our adjusted analyses, we found that younger age was associated with increased odds of presenting to the ED vs an outpatient clinic, as was living in the North-Central or South US, living in an urban area, and being a dependent rather than the primary policy holder (eTable 2 in Supplement 1). Having established prenatal care and prior diagnosis of infertility or threatened EPL were associated with lower odds of presenting to the ED. The presence of a medical comorbidity also remained significantly associated with ED presentation in the adjusted analysis.

Primary management differed significantly between the ED and outpatient groups. Of those seen initially in the outpatient setting, 23 588 patients (24.3%) underwent surgical management (vs 2925 patients [14.0%] seen in the ED) and 10 878 patients (11.2%) received medication management (vs 1116 patients [5.4%] in the ED setting).

EPL management remained significantly associated with location of initial EPL diagnosis when controlling for demographic characteristics, prior pregnancy care, and comorbidities (Table 2). Presenting initially to the ED (adjusted odds ratio [aOR], 0.46; 95% CI, 0.44-0.47), urban location (aOR, 0.87; 95% CI, 0.82-0.91), and being a dependent on an insurance policy (vs primary policy holder) (aOR, 0.71; 95% CI, 0.67-0.74) were associated with decreased odds of receiving active management. Factors associated with increased odds of active management of EPL included older age (aOR per 1-year increase 1.01; 95% CI, 1.01-1.01) or living outside of the West coast (eg, Northeast: aOR, 1.20; 95% CI, 1.15-1.25). A history of infertility (aOR, 0.80; 95% CI, 0.76-0.84) or threatened EPL earlier in the pregnancy (aOR, 0.69; 95% CI, 0.67-0.71) was associated with decreased odds of active management, whereas having established prenatal care (aOR, 2.35; 95% CI, 2.29-2.42) and the presence of medical comorbidities (aOR, 1.05; 95% CI, 1.02-1.09) were both associated with increased odds of active management.

**Table 2.** Factors Associated With Receiving Surgical or Medication Management vs Expectant Management for Early Pregnancy Loss

Variable	aOR (95% CI)
Presentation to emergency department vs outpatient clinic	0.46 (0.44-0.47)
Age, y	1.01 (1.01-1.01)
Insurance coverage	
Primary or spouse	1 [Reference]
Dependent	0.71 (0.67-0.74)
Metropolitan statistical area	
Rural	1 [Reference]
Urban	0.87 (0.82-0.91)
Geographic region	
Northeast	1.20 (1.15-1.25)
North-Central	1.11 (1.06-1.15)
South	1.24 (1.20-1.28)
West	1 [Reference]
Established prenatal care in current pregnancy	2.35 (2.29-2.42)
Threatened early pregnancy loss in current pregnancy	0.69 (0.67-0.71)
History of infertility diagnosis or treatment	0.80 (0.76-0.84)
History of comorbidity	1.05 (1.02-1.09)

Abbreviation: aOR, adjusted odds ratio.

Although the risk of complications was low overall, patients seen in the ED were more likely to require a blood transfusion (287 patients [1.4%] vs 202 patients [0.2%]), require additional surgery including laparoscopy or laparotomy, and have a diagnosis of intrauterine infection (Table 3). Patients in the ED cohort were also more likely to be hospitalized in the subsequent 6 weeks (463 patients [2.2%] vs 472 patients [0.5%]), both for any reason and for EPL-related indications. Our composite complication outcome occurred at a higher proportion in the ED vs outpatient group (726 patients [3.5%] vs 756 patients [0.8%]). In multivariable logistic regression, factors associated with increased odds of composite complication were presentation to the ED vs outpatient clinic (aOR, 6.23; 95% CI, 5.20-7.46), the presence of at least 1 medical comorbidity (aOR, 1.87; 95% CI, 1.56-2.26), and receiving primary surgical management vs expectant management (aOR, 9.34; 95% CI, 7.72-11.3). Receiving medication management vs expectant management was associated with decreased odds of having a composite complication (aOR, 0.73; 95% CI, 0.56-0.95).

**Table 3.** Complications Within 6 Weeks Following Early Pregnancy Loss Management

Complication	Participants, No. (%)			*P* value
	Total (N = 117 749)	ED cohort (n = 20 826)	Outpatient cohort (n = 96 923)	
Inpatient hospitalization				
Any reason	1322 (1.1)	563 (2.7)	759 (0.8)	<.001
Early pregnancy loss related	935 (0.8)	463 (2.2)	472 (0.5)	<.001
Hemorrhage requiring blood transfusion	489 (0.4)	287 (1.4)	202 (0.2)	<.001
Uterine artery embolization	7 (<0.1)	3 (<0.1)	4 (<0.1)	.08
Hysterectomy	30 (<0.1)	3 (<0.1)	27 (<0.1)	.27
Other surgical management (laparotomy or laparoscopy)	83 (0.1)	25 (0.1)	58 (0.1)	.003
Cervical injury or laceration repair	20 (<0.1)	2 (<0.1)	18 (<0.1)	.37
Intrauterine infection	523 (0.4)	192 (0.9)	331 (0.3)	<.001
Uterine perforation or other genitourinary injury	39 (0.1)	3 (<0.1)	36 (<0.1)	.10
Repeat uterine aspiration, No. of participants/total No. (%)^a^	2461/26 513 (9.3)	617/2925 (21.1)	1844/23 588 (7.8)	<.001
Composite complication variable^b^	1482 (1.3)	726 (3.5)	756 (0.8)	<.001

Abbreviation: ED, emergency department.

^a^ The denominator is the subgroup that underwent surgical management initially.

^b^ The composite complication variable included hospitalizations related to early pregnancy loss, blood transfusion, and hysterectomy or other surgical management (apart from uterine aspiration).

We conducted sensitivity analyses with the patients who presented to both an ED and an outpatient clinic on the same calendar day as their initial EPL diagnosis. These 975 patients were excluded from the primary analysis because it was not possible to determine whether they presented to the ED or outpatient clinic first. The management profile of this combined presentation group was significantly different from both the ED group and the outpatient group, with 366 patients (37.5%) undergoing surgical management, 96 patients (9.9%) receiving medication management, and 513 patients (52.6%) receiving expectant management. They were also more likely to experience complications, with 16 patients (1.6%) receiving a blood transfusion, 22 patients (2.3%) being admitted for EPL-related reasons, and 38 patients (3.9%) having one of the composite complications. None of our regression analysis results was affected by including this combined ED and outpatient group with either the ED cohort or the outpatient cohort.

## Discussion

In this cohort study of a large sample of privately insured patients, EPL care differed between ED and outpatient settings. Consistent with our hypothesis, patients with EPL seen in the ED were less likely to receive active management (surgical or medication management) compared with patients seen in the outpatient setting. Complication rates were higher in the ED setting, which may, in part, reflect higher acuity of this patient population, but complication rates were low in both settings. Although it was not possible to determine individual patient preferences for primary EPL management in these data, these differences between the ED and outpatient settings may reflect increased barriers to routine care and inability to access comprehensive EPL management options among patients seeking care in the ED. More research is needed to understand these significant differences in management approaches by location and to what extent EPL management reflects patient preferences in the outpatient and ED settings.

Previous studies^7^ have demonstrated that a large volume of patients (approximately 900 000 in the US annually) present to the ED with EPL-related concerns. Patients who come to the ED with EPL are younger and more likely to be Black or Hispanic compared with other patients in the ED.^7,21^ We similarly found that patients with EPL in the ED were younger, and we also found that they were less likely to be the primary insurance policy holder or to have established prenatal care than patients presenting to the outpatient setting; these characteristics were also all associated with decreased odds of active EPL management. Even when controlling for available demographic and clinical characteristics, presentation to the ED vs outpatient setting for EPL was still associated with decreased odds of receiving active EPL management.

EPL management is a preference-sensitive decision; for patients who are stable, there is clinical equipoise between management options. Outcomes and satisfaction with EPL management have been clearly correlated with a patient’s ability to choose their management option, and shared decision-making is very important in this setting.^22,23^ Patients who may already have decreased access to comprehensive EPL management (because of the aforementioned factors, including younger age and lack of established prenatal care) may experience further barriers to receiving surgical or medication management owing to their greater likelihood of receiving care in the ED. It remains to be determined what role this plays in the dissatisfaction seen among patients who receive EPL care in the ED.

### Limitations

This study has some limitations. Our study is limited by the accuracy of billing codes related to EPL diagnosis and management, and EPL in particular is often underreported and may be diagnosed differently in the ED vs outpatient settings.^24^ Claims data are intended for billing purposes, and billing codes may be incomplete or inaccurate for our exposures and covariates of interest. Our study is based on a large convenience sample of privately insured individuals only. Privately insured individuals account for approximately 62% of the US population, and are disproportionately White and have higher income.^25,26^ To ensure the generalizability of our findings and identify any greater disparities in care that may be uncovered in a broader population, it would be helpful to repeat these analyses in a data set that includes patients with public insurance or no insurance. Additionally, MarketScan has limited demographic data. Studies have shown that EPL is more common among women from minoritized and low-income populations, and these populations are more likely to face barriers in accessing routine health care services.^27^ Compared with women presenting to the ED for other reasons, women presenting to the ED for EPL are more likely to be Black or Hispanic.^7^ Further research is needed to know how EPL management differs for patients from minoritized populations in the ED vs outpatient setting.

MarketScan includes outpatient prescription data but lacks data on medications administered on site. It is possible that patients were given mifepristone or misoprostol in the ED, and this information is not available in our data, leading to misclassification of medication management as expectant management. Conversely, it is possible that some patients were prescribed misoprostol for another indication, such as management of retained products of conception following a completed spontaneous abortion. Additionally, we are unable to determine reliably which patients received in-person consultations from, or were seen in ED facilities with access to, on-call obstetrician-gynecologists. MarketScan also lacks detail regarding individual patients’ hemodynamic stability or acuity, and other unmeasured indicators of severity may confound our results. We do not know the proportion of patients for whom surgical management was clinically indicated over medication or expectant management.

## Conclusions

In this cohort study, patients with EPL who presented to the ED were younger and less likely to have established prenatal care; they may also need additional help and resources compared with patients who presented first to an outpatient setting. At the same time, rates of intervention (surgical and medication management) were lower in the first week following an initial diagnosis in the ED vs an outpatient clinic. Further research is needed to understand whether patients presenting to the ED with EPL are receiving their preferred management option via shared decision-making and to identify any barriers to providing patient-centered EPL care in the ED.

## Notes

Supplement 1. **eTable 1.** Diagnosis and Procedure Codes **eTable 2.** Factors Associated With ED Versus Outpatient Treatment

Supplement 2.
Data Sharing Statement
