# Supplementary material for: Early Pregnancy Loss Management in the Emergency Department vs Outpatient Setting
Source: JAMA Netw Open. 2023 Mar 15;6(3):e232639. doi: 10.1001/jamanetworkopen.2023.2639 (PMC10018323; doi:10.1001/jamanetworkopen.2023.2639)
Supplement: Supplement 1. — eTable 1. Diagnosis and Procedure Codes eTable 2. Factors Associated With ED Versus Outpatient Treatment [file jamanetwopen-e232639-s001.pdf]

Supplemental Online Content

Benson LS, Holt SK, Gore JL, et al. Early pregnancy loss management in the emergency department vs outpatient setting. *JAMA Netw Open*. 2023;6(3):e232639. doi:10.1001/jamanetworkopen.2023.2639

eTable 1. Diagnosis and Procedure Codes
eTable 2. Factors Associated With ED Versus Outpatient Treatment

This supplemental material has been provided by the authors to give readers additional information about their work.

© 2023 Benson LS et al. *JAMA Network Open.*

eTable 1. Diagnosis and Procedure Codes

	ICD-10 diagnosis codes	CPT codes	ICD-10 procedure codes
Inclusion Criteria			
Early pregnancy loss (EPL)	O02.1, O03.X		
Exclusion criteria			
Ectopic or molar pregnancy	O00.X, O01.X, O08.X		
Induced abortion	O04.X, Z33.2	59840, 59841, 59851	
Stillbirth	P95.X, Z37.1, Z37.4, Z37.7, O36.4, O36.4XX0, O36.4XX1, O36.4XX3, O36.4XX4, O36.4XX5, O36.4XX9		
Recent medication management of EPL	*Based on NDC codes for mifepristone and misoprostol*		
Recent surgical management of EPL		59820, 59812, 59821	0UDBxxx
Primary outcome			
Surgical management		59820, 59812, 59821	0UDBxxx
Medication management	*Based on NDC codes for mifepristone and misoprostol*		
Complications			
Inpatient hospitalization, EPL-related	O02.1, O03.X, N93.8, N93.9, O20.x		
Hemorrhage requiring blood transfusion		36430	30233xx
Uterine artery embolization		37241, 37243, 37244	04LE3xx
Other surgical management (laparoscopy or laparotomy)		49320, 49000, 58578	0WJGxxx, 0UQ9xxx
Cervical injury or laceration repair	O71.3	57720	0UQCxx
Uterine perforation or other GU injury	N99.7, N99.71		
Infection related to EPL	O03.0, O03.37, O03.5, O03.87		
Additional covariates			
Prior threatened abortion or bleeding in early pregnancy	O20.X, O26.85X		
Prenatal care in current pregnancy	Z34.X, O09.X		
History of infertility	N97.X, N46.X, O09.81X		

Abbreviations: EPL, early pregnancy loss; ICD, International Classification of Diseases; CPT, Current Procedural Terminology; NDC, National Drug Code; GU, genitourinary

© 2023 Benson LS et al. *JAMA Network Open.*

eTable 2. Factors Associated With ED Versus Outpatient Treatment

	Adjusted odds ratio	95% confidence interval
Age in years	0.95	0.95 – 0.96
Insurance coverage		
Primary or spouse	Reference	
Dependent	1.69	1.60 – 1.78
MSA		
Rural	Reference	
Urban	1.19	1.12 – 1.27
Geographic region		
Northeast	0.95	0.90 – 1.01
North/Central	1.15	1.09 – 1.21
South	1.29	1.23 – 1.35
West	Reference	
Established PNC in current pregnancy	0.90	0.87 – 0.93
Threatened EPL in current pregnancy	0.75	0.73 – 0.78
History of infertility diagnosis or treatment	0.39	0.35 – 0.43
History of comorbidity	1.91	1.84 – 1.98

Abbreviations: ED, emergency department; MSA, metropolitan statistical area; PNC, prenatal care; EPL, early pregnancy loss

© 2023 Benson LS et al. *JAMA Network Open.*
