# Supplementary material for: Early Pregnancy Loss Management in the Emergency Department vs Outpatient Setting
Source: JAMA Netw Open. 2023 Mar 15;6(3):e232639. doi: 10.1001/jamanetworkopen.2023.2639 (PMC10018323; doi:10.1001/jamanetworkopen.2023.2639)
Supplement: Supplement 2. — Data Sharing Statement [file jamanetwopen-e232639-s002.pdf]

Data Sharing Statement

Benson. Early Pregnancy Loss Management in the Emergency Department vs Outpatient Setting. *JAMA Netw Open*. Published March 15, 2023.
doi:10.1001/jamanetworkopen.2023.2639

Data
Data available: No

Additional Information
Explanation for why data not available: This study represents a retrospective analysis of insurance claims data; primary data were not collected for the purposes of this study.
